# Objective and Subjective Vision Abilities and Financial Exploitation Among Older Adults

**DOI:** 10.1093/geroni/igaf122.938

**Published:** 2025-12-31

**Authors:** Nava Mironi, Gali Weissberger, Yoav Bergman

**Affiliations:** Bar-Ilan University, Ramat Gan, HaMerkaz, Israel; Bar-Ilan University, Ramat Gan, HaMerkaz, Israel; Ashkelon Academic College, Ashkelon, HaDarom, Israel

## Abstract

Financial exploitation (FE) of older adults, including frauds and scams, has significant negative social/health implications for older adults. Thus, identifying risk factors of FE is imperative for preventing FE and enhancing older adults’ well-being. Subtle visual cues in fraudulent transactions (e.g., emails, websites) may be missed in those who have reduced visual abilities. Poor vision may also increase dependence, thereby intensifying FE vulnerability. However, to our knowledge, no study has examined the relationship between objective/subjective vision abilities and FE. Accordingly, we examined the relationship between vision abilities and FE vulnerability among 97 community-dwelling older adults (M = 70.26, SD = 5.61). Participants completed self-report questionnaires assessing subjective vision difficulties, FE vulnerability, and history of FE experiences. Additionally, participants underwent objective vision assessments (near, distant, and contrast sensitivity) conducted by a certified gerontological optometrist. Multiple linear regression and logistic regression models regressed FE vulnerability and FE, respectively, on subjective and objective vision abilities, covarying for background characteristics. While self-reported vision difficulties were associated with FE vulnerability (β =.355, p<.001) and FE (OR = 7.866, p= .005), objective measures of vision were not. Findings suggest that self-perceived vision difficulties/declines are more strongly associated with FE vulnerability and FE experiences than similar objective measures. It is possible that subjective vision reflects a person’s cognitive averaging of vision ability that is not fully captured in well-controlled environments characteristic of objective assessments. These findings advance our understanding of FE risk factors and suggest practical approaches for identifying and supporting at risk older adults.

